# Identification of Metabolites and Transcripts Involved in Salt Stress and Recovery in Peanut

**DOI:** 10.3389/fpls.2018.00217

**Published:** 2018-02-22

**Authors:** Feng Cui, Na Sui, Guangyou Duan, Yiyang Liu, Yan Han, Shanshan Liu, Shubo Wan, Guowei Li

**Affiliations:** ^1^Biotechnology Research Center, Shandong Academy of Agricultural Sciences, Jinan, China; ^2^Shandong Provincial Key Laboratory of Crop Genetic Improvement, Ecology and Physiology, Jinan, China; ^3^College of Life Science, Shandong Normal University, Jinan, China; ^4^School of Life Sciences, Qilu Normal University, Jinan, China

**Keywords:** metabolomics, peanut, recovery, salt stress, transcriptomics, correlation analysis

## Abstract

**HIGHLIGHTS**

- Metabolites and transcripts related to plant physiology in salt stress conditions, especially to the recovery process were disclosed in peanut.

Peanut (*Arachis hypogaea* L.) is considered as a moderately salt-sensitive species and thus soil salinity can be a limiting factor for peanut cultivation. To gain insights into peanut plant physiology in response to salt stress and alleviation, we comprehensively characterized leaf relative electrolyte leakage (REC), photosynthesis, leaf transpiration, and metabolism of plants under salt stress and plants that were subjected to salt stress followed by salt alleviation period. As expected, we found that REC levels were higher when plants were subjected to salt stress compared with the untreated plants. However, in contrast to expectations, REC was even higher compared with salt treated plants when plants were transferred from salt stress to standard conditions. To decipher REC variation in response to salt stress, especial during the recovery, metabolite, and transcript variations were analyzed by GC/MS and RNA-seq method, respectively. Ninety two metabolites, among total 391 metabolites identified, varied in response to salt and 42 metabolites responded to recovery specially. Transcriptomics data showed 1,742 in shoots and 3,281 in roots transcript varied in response to salt stress and 372 in shoots and 1,386 transcripts in roots responded specifically to recovery, but not salt stress. Finally, 95 transcripts and 1 metabolite are indicated as candidates involved in REC, photosynthesis, transpiration, and Na^+^ accumulation variation were revealed by using the principal component analysis (PCA) and correlation analysis. This study provides valuable information on peanut response to salt stress and recovery and may inspire further study to improve salt tolerance in peanut germplasm innovation.

## Introduction

Peanut (*Arachis hypogaea* L.), as an important oil and food crop, is widely grown in tropical, subtropical, and temperate region in the world and mainly cultivated in Asia, Africa, and Americas. In China, to gain more crop production, low-salinity fields were attempted to apply for peanut cultivation in recent years, although peanut is considered as a moderately salt-sensitive species (Singh et al., 2008; Chakraborty et al., 2016). The irrational irrigation also cause the soil affected by secondary salinity (Munns and Tester, 2008). Peanut growth in these types of fields will be impaired by salinity in certain extent depending on the soil water content. Furthermore, plants will be suffered to too rapid rehydration from the salt relief after heavy rain in summer. The ability for plants to recover under abiotic stresses is also important when the stress was relieved, which was also worth more emphases as done in salt stress field (Zhu, 2002; Gechev et al., 2012). Studies about the involved mechanisms will favor our understanding of how the peanut adapts to salt stress environment.

It has been reported that salinity can decrease peanut seed germination and seedling growth (Janila et al., 1999; Mensah et al., 2006; Singh and Prasad, 2009; Salwa et al., 2010), inhibit photosynthesis (Qin et al., 2011), induce Na^+^ accumulation, and Ca^2+^, K^+^, and Mg^2+^ deficiency (Taffouo et al., 2010) and result in the accumulation of organic metabolites (Parida and Jha, 2013). There is also evidence that external K^+^ application could improve salinity tolerance (Chakraborty et al., 2016). Some peanut genes have been reported to be function in salt stress response. For instance, drought-induced peanut AhNAC2 overexpressed transgenic *Arabidopsis* exhibited enhanced tolerance to drought and salinity stress (Liu et al., 2011). Overexpression of peanut allene oxide cyclase (AhAOC) in rice conferred tolerance against salinity (Liu et al., 2015). However, the researches about salt stress response genes in peanut are rare up to now.

Plant metabolites, serving as osmolytes and osmoprectants, involved in biotic and abiotic stress response under salt stress, drought, and desiccation stress (Shulaev et al., 2008). Metabolism is considered as a powerful tool to study plant physiology in response to abiotic stresses and metabolites are closer to phenotype compared to genes and proteins, more accurately reflecting the comprehensive results of gene expressions and various regulatory processes (Scherling et al., 2010; Arbona et al., 2013; Ramalingam et al., 2015). However, peanut metabolites in response to salt stress are still unclear.

To identify the metabolites and transcripts of peanut involved in salt stress and especially during the recovery conditions, we comprehensively characterized peanut physiology and metabolite production of plants under salt stress and relieved conditions. And powerful RNA-seq method was employed to analyze key salt stress response genes. By transcriptome analysis, 70,215 unigenes including 55,846 coding sequences (CDSs) were identified. And 1,742 varied transcripts in shoots and 3,281 in roots in response to salt stress (N4), respectively. And 372 and 1,386 transcripts were identified specific response to R3 (salt alleviation) in shoots and roots, respectively. Finally, the correlation of physiology parameters, metabolite production, and gene expression were analyzed and the metabolites and transcripts related to plant physiology variation were revealed. This study provides plenty valuable information about how peanut responds to salt stress and disclosed the transcripts related to the stress alleviation.

## Materials and methods

### Plant cultures and salt-stress treatments

The peanut cultivar Luhua 14 (LH14) was used as the experimental material in this study. After germination in sand for 8 days, peanut seedlings were transferred to hydroponic pots containing 2 L of Hoagland's nutrient solution and grown in an artificial climate-controlled chamber with 16 h light (200 μmol protons m^−2^ s^−1^, 26°C) and 8 h darkness (24°C) at 50% relative humidity. The nutrient solution was changed weekly. NaCl treatments, each containing four replicate plants in separate pots, were as follows: Hoagland's nutrient solution with addition of 0 (Standard conditions, ST), 50, 100, 150, 200, 250, 300, or 400 mM NaCl. Treatment began when seedlings were 18 days old. After 4 days (N4), the salt-stress recovery treatment was transferred back to standard Hoagland's solution for 3 days (R3). At the end of the experiment, shoots and roots were collected, immediately frozen in liquid nitrogen, and stored at −80°C until analysis.

### Plant physiological analysis

Electrolyte leakage was analyzed as described previously (Li et al., 2008). Briefly, leaves were washed in deionized water and cut into 1 cm slices and then immersed in 5 mL deionized water in a test tube at room temperature for 2 h. Tubes were set in boiling water for 10 min and then cooled to room temperature. The relative electrical conductivity (REC) was calculated from the electrical conductivity of the solution before and after boiling.

Leaf net photosynthesis and transpiration rate were measured using a portable photosynthetic system (CIRAS-2, PP Systems, Hitchin, Hertfordshire, UK) under ambient CO_2_ concentration (370 μmol mol^−1^), a PPFD of 800 μmol m^−2^ s^−1^, leaf temperature of 26 ± 1°C and relative air humidity of 50% (Zhang et al., 2010).

Sodium and potassium content measurements were performed as described previously (Kong et al., 2012). Leaves and roots samples were immersed in 0.1 M HCl overnight. Concentrations of Na^+^ and K^+^ were determined using an atomic absorption spectrophotometer (TAS-990, Beijing, China).

### Metabolite extraction and analysis

The metabolite extraction and analysis were performed using gas chromatography-mass spectrometer (GC-MS) as described previously (Lisec et al., 2006; Fernie et al., 2011). Briefly, peanut shoots and roots were quick excision and snap-freezing in liquid nitrogen, and stored at −80°C. Then the samples were ground into powder in liquid N_2_, and 60 mg dried powder was bathed in a cooled water-methanol-chloroform mixture (2:2:1) with L-2-chloro-phenylalanine as internal standard and sonicated for 40 min. After centrifugation at 14,000 rpm for 10 min, 700 μL supernatant was dried and reacted with 80 μL methoxamine hydrochloride at 37°C for 90 min, followed by a silylation reaction. Each 1 μL aliquot of the derivatized solution was injected in splitless mode into the Agilent 7890A-5975C GC-MS system (Agilent, USA). Separation was carried out on a non-polar DB-5 capillary column (30 m × 250 μm I.D., J&W Scientific, Folsom, CA), with high purity helium as the carrier gas at a constant flow rate of 1.0 mL/min. The GC temperature programming began at 60°C, followed by 8°C/min oven temperature ramps to 125°C, 4°C/min to 210°C, 5°C/min to 270°C, and 10°C/min to 305°C, and a final 3 min maintenance at 305°C. The electron impact (EI) ion source was held at 260°C with a filament bias of −70 V. Full scan mode (m/z 50–600) was used, with an acquisition rate of 20 spectrum/second in the MS setting.

The acquired MS data were analyzed by ChromaTOF software (v4.34, LECO, St Joseph, MI, USA) according to Kind et al. (2009). Briefly, after alignment with Statistic Compare component, the CSV file was obtained with three dimension data sets including sample information, retention time-m/z, and peak intensities, The detectable peaks of plant samples in GC-MS were 355/490 in total, and the internal standard was used for data quality control (reproducibility). After internal standards and any known pseudo positive peaks, such as peaks caused by noise, column bleed, and BSTFA derivatization procedure, were removed from the data set, and the peaks from the same metabolite were combined. The data set was normalized using the sum intensity of the peaks in each sample.

The data sets resulting from GC-MS were separately imported into SIMCA-P+ 14.0 software package (Umetrics, Umeå, Sweden). Principle component analysis (PCA) and (orthogonal) partial least-squares-discriminant analysis (O)PLS-DA were carried out to visualize the metabolic alterations among experimental groups, after mean centering and unit variance scaling. Variable importance in the projection (Variable Importance in Projection, VIP) ranks the overall contribution of each variable to the (O)PLS-DA model, and those variables with VIP > 1.0 are considered relevant for group discrimination. All of the differentially expressed compounds in treated group were selected by comparing the compounds in the treated group with the control using the multivariate statistical method and Wilcoxon-Mann-Whitney test. Metabolites with both multivariate and univariate statistical significance (VIP > 1.0 and *p* < 0.05) were annotated with the aid of available reference standards and the NIST 11 standard mass spectral databases and the Feihn database linked to ChromaTOF software.

For this analysis, three independent biological replicates of shoot and root samples and two technical replicates were performed. The relative abundance of metabolites was presented as the average values of the six replicates. A Student's *t*-test with *p*-value < 0.05 was used for metabolite variation identification. The raw data was submitted to MetaboLights database with an identification number MTBLS583.

### Sample preparation and total RNA extraction

Frozen samples were ground in liquid nitrogen. For roots, total RNA was extracted using TRIzol reagent; for shoots, total RNA was extracted by the CTAB method (Song et al., 2016). The RNA quality and quantity were determined using an Agilent 2100 Bioanalyzer (Agilent Technologies). For the transcriptome sequencing, equal amount of RNA from each sample was mixed. All the shoot and root samples have three independent biological replicates in ST, N4, and R3 conditions, respectively.

### Transcriptome sequencing and data analysis

For transcriptome sequencing, RNA treatment, library construction, and deep sequencing were carried out as described previously (Li et al., 2013). Briefly, a cDNA library was prepared by mixing equal amount of RNA from shoots and roots samples in ST, N4, and R3 together. After the total RNA extraction and DNase I treatment, magnetic beads with Oligo (dT) are used to isolate mRNA. Mixed with the fragmentation buffer, the mRNA is fragmented into short fragments. Then cDNA is synthesized using the mRNA fragments as templates. Short fragments are purified and resolved with elution buffer for end reparation and single nucleotide A (adenine) addition. After that, the short fragments are connected with adapters. The suitable fragments are selected for the PCR amplification as templates. During the QC steps, Agilent 2100 Bioanaylzer and ABI StepOnePlus Real-Time PCR System are used in quantification and qualification of the sample library. The libraries were sequenced using Illumina HiSeq™ 2000 at the Beijing Genomics Institute (BGI). After sequencing, raw reads were acquired, and then clean reads were obtained by removing reads with adapters and reads with unknown nucleotides lager than 5%, and low quality reads. The clean reads were used for *de novo* assembly which is carried out with the program Trinity (Zhao et al., 2011). Trinity partitions the sequence data into many individuals de Bruijn graphs, each representing the transcriptional complexity at a given gene or locus, and then processes each graph independently to extract full-length splicing isoforms and to tease apart transcripts derived from paralogous genes. The result sequences of trinity were called unigenes. Unigenes from each sample's assembly were further processed with sequence clustering software to acquire non-redundant unigenes as long as possible. After clustering, the unigenes were divided into clusters (CL prefix) and singletons (Unigene prefix). Data analysis was according to Xia et al. (2013). Unigenes were first aligned by BlastX (E value < 0.00001) to protein databases in the priority order of NR (non-redundant database) (ftp://ftp.ncbi.nlm.nih.gov/blast/db), Swiss-Prot (Boutet et al., 2007), KEGG (Kyoto Encyclopedia of Genes and Genomes) (Kanehisa et al., 2008), and COG (Clusters of Orthologous Groups of proteins) (Roman et al., 2000). Proteins with the highest ranks in the blast results were taken to decide the coding region sequences of unigenes, and the coding region sequences were translated into amino sequences with the standard codons. Thus, we acquired both the nucleotide sequences (5′−3′) and the amino acid sequences of the unigene coding region. Unigenes that could not be aligned to any database were scanned by ESTScan, producing nucleotide sequence (5′−3′) direction and the amino acid sequence of the predicted coding region (Iseli et al., 1999).

### Identification and functional annotation of different expression genes (DEGs)

To characterize DEGs response to salt stress and recovery in peanut, both shoots and roots in N4 and R3 were analyzed by RNA-seq analysis via Ion Proton^TM^ system (Life Technologies) to identify DEGs (Riera et al., 2017). Six tag libraries were constructed for both shoots and roots in ST, N4, and R3 conditions. To check the homogeneity of the biological samples, cluster tree of all samples was performed. The distances of expressed gene in samples are calculated by the Euclidean method. Meanwhile, the algorithm of sum of squares of deviations was used to calculate the distance between samples. The samples for both shoots and roots in standard, salt stress and recovery conditions were clustered respectively. NOISeq method was used to screen differentially expressed genes between three biological samples using the criteria: |log2ratio| ≥ 1 and probability ≥ 0.8 (Tarazona et al., 2011). For gene expression profiling, Gene Ontology (GO) enrichment analysis of functional significance was applied using the hypergeometric test to map all differentially expressed genes to terms in the BGI WEGO (Web Gene Ontology Annotation Plotting, http://wego.genomics.org.cn/) looking for significantly enriched GO terms in the differentially expressed genes, and comparing them to the transcriptome database. To identify DEGs across the samples, all clean tags were mapped to the reference sequences allowing 1 bp mismatch. The clean tags mapped to reference sequences from multiple genes were filtered. The remaining clean tags were designed to be unambiguous. The number of unambiguous clean tags for each gene was calculated and normalized to TPM (the number of transcripts per million clean tags) (Morrissy et al., 2009). The statistical enrichment of DEGs in KEGG pathways was analyzed by using KOBAS software according to Mao et al. (2005). Rich factor represents enrichment intensiveness, which was calculated from the ratio of the DEGs number and the number of genes annotated in a certain pathway.

The raw data of transcriptome and RNA-Seq has been deposited in NCBI Sequence Read Archive (SRA, https://www.ncbi.nlm.nih.gov/sra) with the Bioproject number PRJNA398720. This Transcriptome Shotgun Assembly (TSA) project has been deposited at DDBJ/EMBL/GenBank with the accession number GGBX01000000. Each data package represents a biological replicate from shoots or roots in ST, N4, and R3 conditions, respectively.

### Real-time quantitative RT-PCR (q-PCR)

The transcripts abundance were investigated as described (Sutka et al., 2011). Briefly, total RNA was extracted as described as sample preparation for RNA-seq. The cDNA was synthesized using PrimeScriptTM RT reagent Kit with gDNA Eraser (TaKaRa, RR047A). To control the genomic DNA contamination of mRNA, CL1315.Contig1 was uses as a target gene by PCR with left primer (CGGAAATGGCGGACAAGTACAACC) and right primers (TCAAATTCAGTATCACGAGGCCCC). The PCR product length was 169 and 749 bp from cDNA and genomic DNA, respectively. The sequences of primer pairs used for gene-specific amplification of the 22 genes and seven reference genes are listed in Table S8. To identify stable reference genes under salt treatments and recovery in roots and shoots, several transcripts with little variation under the treatment conditions or homologs of frequently used reference genes in Arabidopsis including RAD23 (Unigene14502), EF1α (Unigene13604), tubulin α (CL11012.Contig1), actin (Unigene21166), EF1β (Unigene17338), F-box protein (Unigene20560), uncharacterized protein (Unigene817) were investigated and their expression stability was analyzed with geNORM version 3.4 software (Vandesompele et al., 2002). For each gene investigated, PCR efficiency (E) was calculated and used to quantify gene expression as described (Postaire et al., 2010). Finally, CL11012.Contig1 and Unigene817 in shoots and Unigene14502 and Unigene20560 in roots were selected as the reference genes in salt stress and recovery conditions.

### Statistical analysis

Variations among treatments of physiological data were examined using one-way ANOVA and Tukey multiple comparison test, which were done by using PRISM software (version 4.02; GraphPad software Inc, San Diego, CA). The R statistical language (http://www.r-project.org/) was used for most of the statistical analyses in this study. Log2 normalization was applied before statistical analysis. The influence of treatment in the experiment upon individual metabolite/transcript/physiological index was checked by one-way ANOVA with the false discovery rate (FDR) correction (Benjamini and Hochberg, 1995). Principal component analysis (PCA) was done using R package “pcaMethods”. R function “cor.test” in “stats” package was used for Pearson correlation analysis (Stacklies et al., 2007; Heyneke et al., 2017).

## Results

### The physiological changes of peanut seedlings after salt stress and recovery

**Figure 1:**
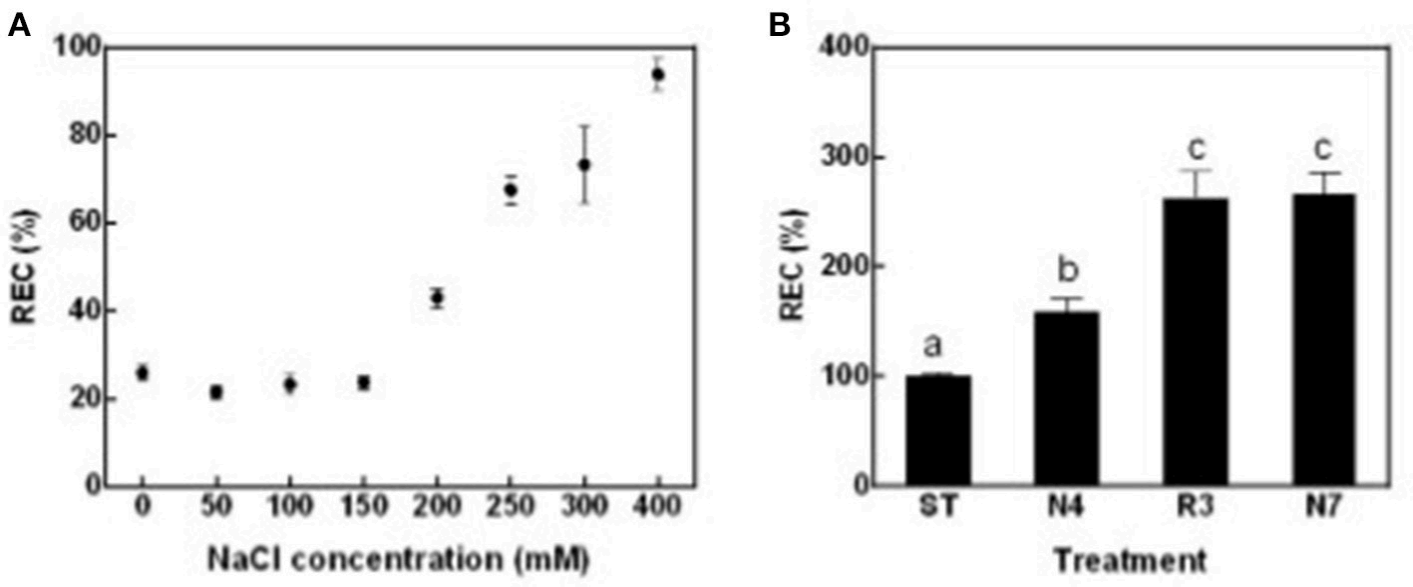
Leaf relative electric conductivity (REC) of peanut seedlings grown with various treatments. **(A)** The RECs of peanut seedlings under different NaCl concentration. Eighteen-day-old seedlings treated with 0, 50, 100, 150, 200, 250, 300, and 400 mM NaCl for 4 days. Data were pooled from at least two independent plant cultures with *n* ≥ 12 at each point. **(B)** The RECs of peanut seedlings in standard conditions (ST) and treated with 250 mM NaCl for 4 days (N4) or 7 days (N7), and recover from N4 for 3 days (R3). Data were pooled from three independent repeats with *n* ≥ 16 at each point. RECs (mean ± SE) were expressed as percentage of mean REC of plant grown in ST conditions. Different letters above the bars indicate significant difference at *p* < 0.05 as determined by one-way ANOVA followed by Tukey's test.

To globally evaluate impacts of salt stress and following salt alleviation on peanut physiology, leaf relative electric conductivity (REC), Na^+^ and K^+^ concentration, net photosynthesis rate (Pn), and transpiration rate (Tr) were measured. Leaf REC, which reflects plasma membrane damage and electrolyte leakage, was significantly higher in plants treated with salt concentrations that exceeded 200 mM NaCl compared with seedlings in standard conditions without supplemental salt (ST) (Figure 1A). According to these data, 250 mM NaCl for 4 days (N4) were chosen as salt treatment in all the following experiments. In contrast to expectations, peanut seedlings growth did not improve, but more leaves grew yellow and died when transferred back to ST conditions after being in N4 conditions (data not shown). REC values of plants in the recovery treatment (R3) were even higher than that in N4 and similar to the levels of plants continuously kept in salt stress for 7 days (N7) (Figure 1B). The results suggest that cell membrane damage might not be reversible and that a greater stress was imposed when the high-salt-treated seedlings were transferred directly to ST conditions.

**Figure 2:**
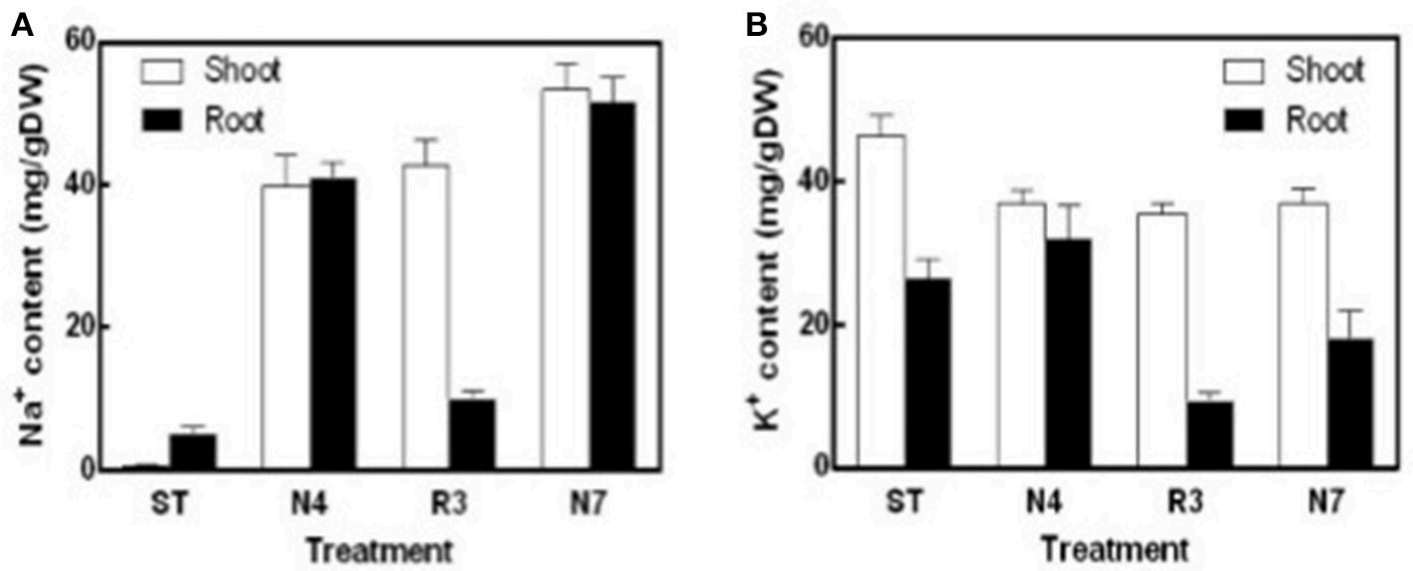
Na^+^ and K^+^ contents of shoots and roots in different treatments. Na^+^ and K^+^ were determined using an atomic absorption spectrophotometer and Na^+^ **(A)** and K^+^ **(B)** content (mg/gDW) were calculated with the shoots or roots dry weight in ST, N4, R3, and N7. Data were pooled from two independent repeats with *n* ≥ 6 at each point and values are means ± SE.

Na^+^ content in both shoots and roots in N4 treatment reached to about 40 mg/g DW, which is 85.6 and 10.5-fold higher compared to that in shoots and roots in ST conditions, respectively. Salt concentrations were even higher in N7 plants, which is 53.27 ± 3.17 mg/g DW in shoots and 51.61 ± 3.02 mg/g DW in roots, respectively. The Na^+^ concentration in R3 plant shoots remained high, whereas the concentration of Na^+^ in roots was close to the plants in ST (Figure 2A). K^+^ concentration in shoots was lower by 20.1% in N4 plants compared with ST plants, and then there was no more variation in R3 and N7. However, in roots there was no variation for K^+^ concentration in N4, but 31.8% decreased in N7. Curiously, K^+^ content in roots, but not in shoots, decreased dramatically in R3 (Figure 2B).

**Figure 3:**
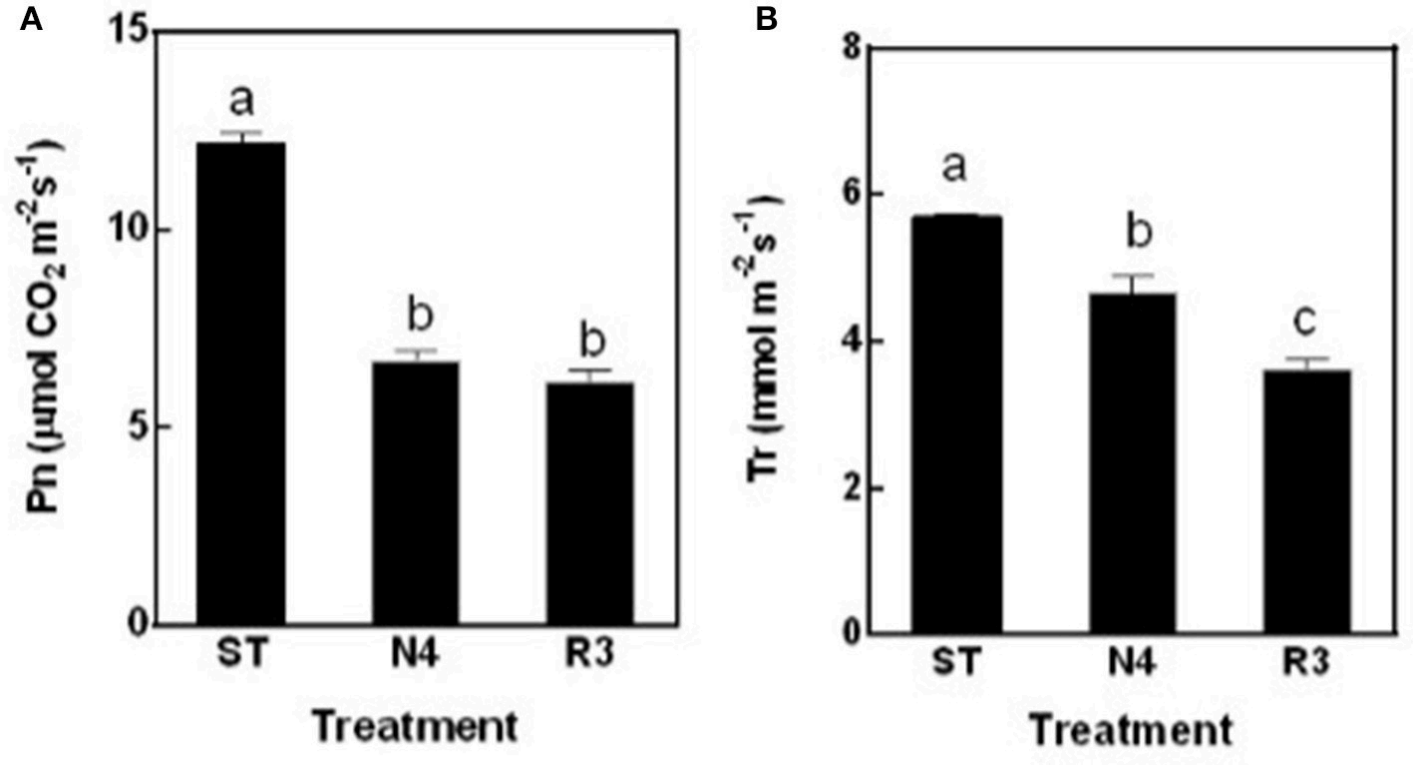
The net photosynthesis rate (Pn) **(A)** and transpiration rate (Tr) **(B)** of peanut leaves were measured in ST, N4, R3, and N7 conditions. Values are means ± SE from at least three independent biological replicates and different letters above the bars indicate significant difference at *p* < 0.05 as determined by one-way ANOVA followed by Tukey's test.

The net photosynthesis rate (Pn) was sharply reduced by 45.1% in N4 compared to ST, but maintained at the similar level in R3 as salt stress (Figure 3A). The transpiration rate (Tr) decreased by 18.3% in N4, but further reduced to 63.4% in R3 compared to that in ST (Figure 3B).

### Metabolite variation in salt stress and recovery

**Figure 4:**
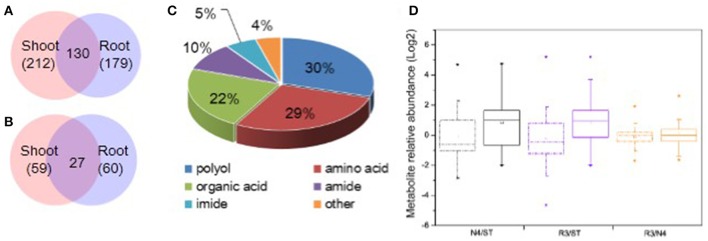
Categorization and variation of metabolites in response to salt stress and recovery. **(A)** All metabolites identified including 212 in shoots and 179 in roots and 130 shared in both tissues. **(B)** Salt stress response metabolites including 59 in shoots and 60 in roots with 27 varied in both tissues. **(C)** Totally, 91 varied metabolites in both shoots and roots were identified including 29.7% polyols, 28.6% amino acids, 22.0% organic acids, 9.9% amides, 5.5% imides, and 4.4% other metabolites. **(D)** Boxplot of salt stress response metabolite variation in N4 and R3 against ST or R3 conditions in shoots and roots, respectively. The dashdot line and solid line box represents data from shoots and roots, respectively. ^*^ and □ represents the extreme and mean values in each conditions, respectively. Data was from metabolite analysis from three biological and two technical replicates.

It is well-known that plant metabolites, serving as osmolytes and osmoprotectant, play important role in abiotic stress response. To identify peanut metabolites involved in the physiological changes during salt stress and recovery, metabolic profiling in both shoots and roots was carried out by GC-MS, respectively. The raw data has been submitted to MetaboLights database and the normalized metabolite data were included in the Table S1. In total, we identified 212 shoot metabolites and 179 root metabolites with 130 common metabolites between shoots and roots (Figure 4A). Of these, 59 shoot metabolites and 60 root metabolites (27 of which were common among treatments) were found at significantly different levels in response to salt stress (Figure 4B). Among all the varied metabolites, polyols, amino acids, organic acids are the major categories, which account for 29.67, 28.57, and 21.98%, respectively (Figure 4C and Tables S1, S2).

Among the salt stress response metabolites, there are larger metabolite variation and much more metabolites increased in roots both in N4 and R3 vs. ST conditions compared that in shoots. However, less metabolite variation was showed in R3 vs. N4 condition in shoots and roots, respectively (Figure 4D). In detail, 34 metabolites were decreased and 25 metabolites were increased in shoots when exposed to salt stress. Among the 59 varied metabolites in salt stress, only nine were recovered to ST conditions and the quantity of 45 metabolites in R3 was between ST and N4 conditions (Tables S1, S2). In roots, 18 metabolites decreased and 42 metabolites increased in salt stress. Among these, only 15 metabolites were found no difference between R3 and ST and the quantity of 40 metabolites in R3 was between ST and N4 conditions (Tables S1–S3).

Among the salt response metabolites, we found that nine metabolites did not have recovery trend but varied in the opposite direction in either shoots or roots, as REC varied in R3 conditions (Table 1). For instance, histidine in roots, which is undetectable in ST conditions, was induced by salt stress and did not decrease, but was accumulated even higher in R3 conditions. Furthermore, we analyzed the metabolites that specially responded to salt stress recovery, but not salt stress. Thirty-five metabolites in shoots but only seven in roots were identified as salt stress recovery specific response metabolites, which might be involved in the process of peanut salt stress recovery (Table S3).

**Table 1:** Metabolites had similar variation pattern with REC in responded to N4 and R3.

**Metabolites**	**Tissue**	**Relative abundance^a^**					
		**ST**	**N4**	**R3**	**N4/ST**	**R3/ST**	**R3/N4**
Urea	Root	29.28 ± 6.23	59.48 ± 6.48	102.15 ± 19.01	2.03^**^	3.49^**^	1.72^*^
Leucine	Root	35.33 ± 5.28	80.82 ± 4.42	135.02 ± 24.01	2.29^**^	3.82^**^	1.67^*^
Uridine	Root	ND^b^	9.81 ± 0.99	41.57 ± 14.36	–	–	4.24^*^
Histidine	Root	ND	34.07 ± 7.13	153.69 ± 25.74	–	–	4.51^**^
L-Malic acid	Shoot	406.96 ± 20.5	95.57 ± 13.53	61.02 ± 8.06	0.23^**^	0.15^**^	0.64^*^
4-Hydroxybutyrate	Shoot	1.82 ± 0.17	0.83 ± 0.07	0.49 ± 0.06	0.46^**^	0.27^**^	0.59^**^
Quinic acid	Shoot	111.14 ± 17.02	56.51 ± 10.89	29.92 ± 4.25	0.51^*^	0.27^**^	0.53^*^
Sedoheptulose	Shoot	3.15 ± 0.24	5.58 ± 0.26	9.17 ± 0.44	1.77^**^	2.91^**^	1.64^**^
Caffeic acid	Shoot	1.29 ± 0.25	2.38 ± 0.37	4.12 ± 0.26	1.84^*^	3.18^**^	1.73^**^

a *Relative abundance of metabolites in ST, N4, and R3 conditions and N4/ST, R3/ST, and R3/N4*.

b *ND, undetected*.

*, ** *mean significant difference by student's t-test with p < 0.05 and p < 0.01, respectively*.

### Differentially expressed genes (DEGs) of peanut in response to salt stress and recovery

To identify genes involved in salt stress and following recover responses, RNA-seq analysis were carried out. Since the genome sequencing of cultivated peanut has not been completed, so the *de novo* transcriptome sequencing was carried out to obtain the precise transcript information of LH14, which will favor transcripts identification. In total, 19,621,058,940 nucleotide (nt) bases were generated and 70,257 unigenes were detected from the assembly database. The average length of all the unigenes was 1,156 nt, and the median was 1,738 nt. For function annotation analysis, the total annotation unigenes were 57,505 and for protein coding region prediction analysis, 55,861 CDSs were identified, which included 53,336 CDSs mapped to the protein databases and 2,525 predicted CDSs (TSA project has been deposit in DDBJ/EMBL/GenBank database). In general, the transcriptome data provided abundant information for subsequent analysis of potential gene regulation in response to salt stress and in recovery conditions of peanut.

**Figure 5:**
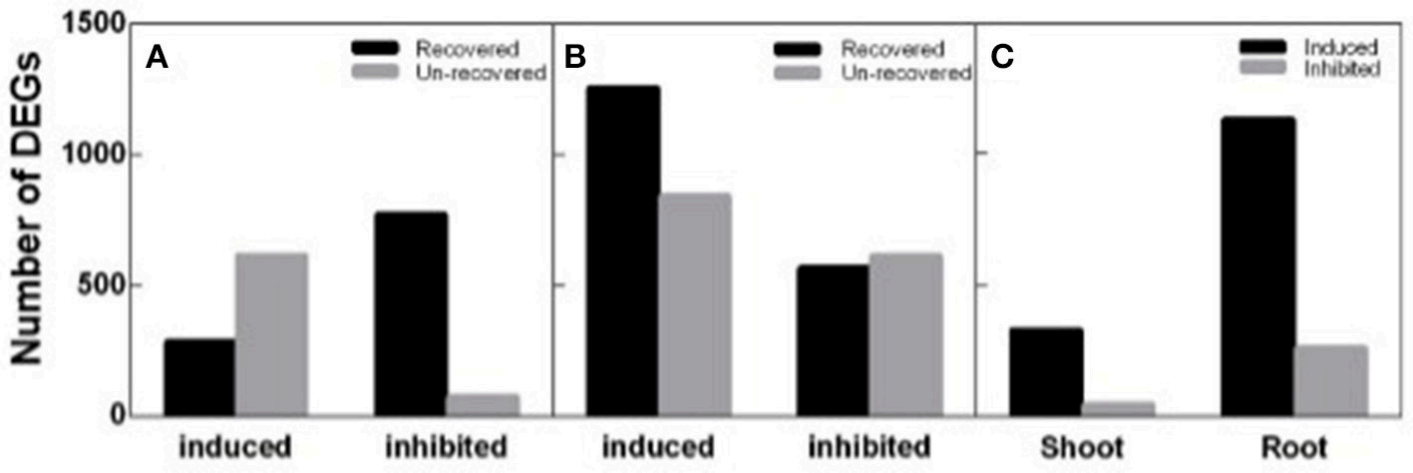
Different expression genes (DEGs) identified in N4 and R3 conditions. Salt stress response genes including salt stress induced and inhibited and the numbers of them recovered or not in R3 conditions in shoots **(A)** and roots **(B)**. **(C)** the number of R3 specific response transcripts including induced and inhibited in shoots and roots.

To identify DEGs response to salt stress and recovery in peanut, both shoot and root transcripts in N4 and R3 were quantified by RNA-seq analysis via Ion Proton platform. Firstly, homogeneity analysis showed that samples in N4 and R3 are clustered in the same group when compared to ST sample for both shoots and roots (Figure S1). Totally, 1,742 transcripts including 898 up-regulated and 844 down-regulated in shoots, and 3,281 transcripts including 2,099 up-regulated and 1,182 down-regulated in roots, responding to salt stress in N4 were identified compared to ST, respectively. In R3 conditions, the expression of 60.51% (1,054) and 44.35% (1,455) transcripts recovered to the ST level in shoots and in roots, respectively. Furthermore, 372 transcripts including 328 up-regulated and 44 down-regulated in shoots and 1,386 transcripts including 1,127 up-regulated and 259 down-regulated in roots specifically responded to R3, but not to salt stress (Figure 5).

**Figure 6:**
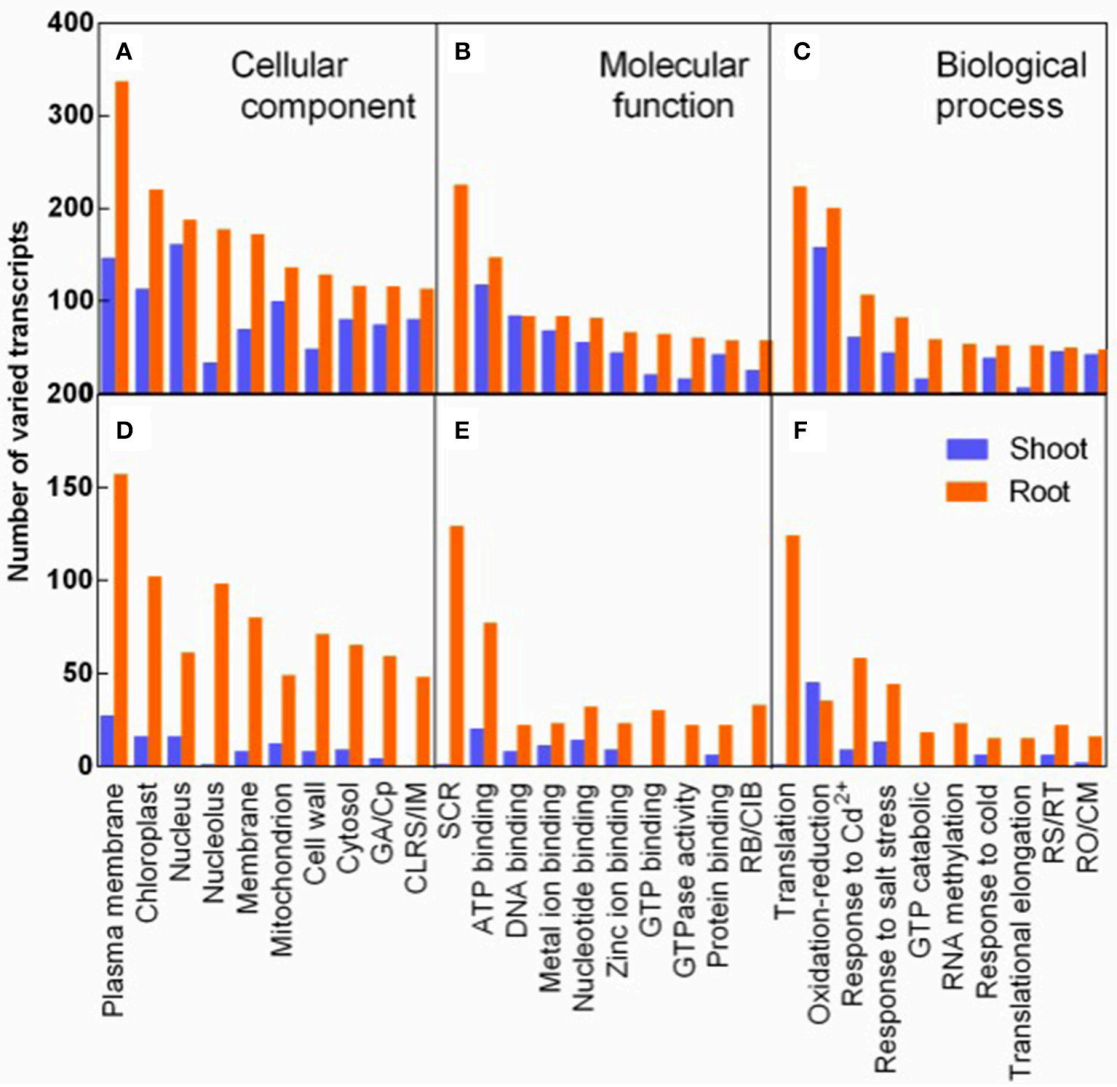
GO classification analysis of DEGs in response to salt stress **(A–C)** and to recovery specifically **(D–F)**. Varied transcripts were categorized into cellular component **(A,D)**, molecular function **(B,E)**, and biological process **(C,F)**. (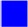) and (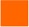) represent varied transcripts in shoots and in roots, respectively. GA/Cp in represents Golgi apparatus term for D/Cytoplasm term for **(A)** in X-axis, respectively. CLRS/IM, Cytosolic large ribosomal subunit/Integral to membrane **(A,D)**; SCR, Structural constituent of ribosome; RB/CIB, RNA binding/Copper ion binding **(B,E)**. RS/RT, Response to stress/Regulation of translation; and RO/CM, Response to oxidative stress/Carbohydrate metabolic process **(C,F)**.

The transcripts varied in response to salt stress and recovery specially were analyzed by GO analysis. The varied transcripts were categorized into cellular component, molecular function, and biological process. In each category, the 10 top largest GO terms were represented both in shoots and in roots (Figure 6). Plasma membrane term was mostly enriched in the cellular component, implying that plasma membrane related transcripts were mostly affected by salt stress (Figure 6A). In the molecular function category, structural constituent of ribosome (SCR) was mostly and specifically enriched in roots. And seven of the 10 most varied terms were various binding terms (Figure 6B). In the biological process category, translation term was mostly and specifically enriched in roots (Figure 6C). The 8-9 GO terms of R3 specific response transcripts were identical to the top 10 largest terms of salt stress responding transcripts in the three categories, although fewer transcripts specifically varied during recovery process (Figures 6D–F).

**Figure 7:**
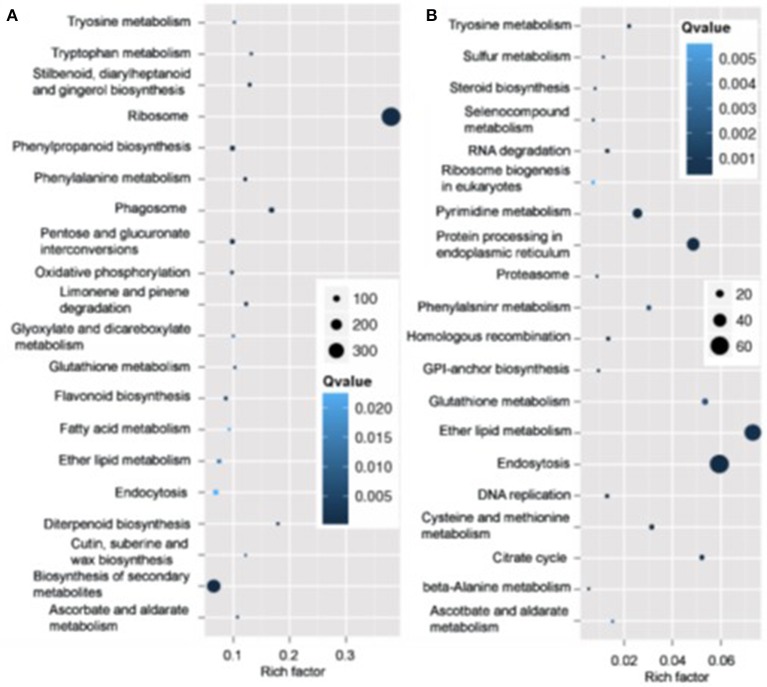
The mostly enriched Kyoto Encyclopedia of Genes and Genomes (KEGG) pathway in roots. **(A)** The top 20 enriched pathway in salt stress conditions (N4 vs. ST) in roots. **(B)** The top 20 enriched pathway in salt stress recovery conditions (R3 vs. N4) in roots. The size and the color of solid circles represent the number of transcripts involved in the certain pathway and the significant value (Qvalue) of the rich factor, respectively.

Pathway analysis, which is a powerful tool to identify involved in biological processes with KEGG database, showed ribosome pathway was mostly enriched and most genes involved in this pathway varied in roots in N4 conditions. However, ether lipid metabolism and endocytosis pathways were the top two most enriched with most transcripts varied in roots (Figure 7). And in shoots, the transcripts of photosynthesis-antenna proteins were mostly enriched and much more transcripts varied in metabolic and biosynthesis of secondary metabolite pathways compared to other pathways in both N4/ST and R3/N4 conditions (Figure S2).

### Salt stress responding gene variations in salt stress and recovery

Among the salt stress response transcripts, 12.5% (218/1742) and 24.3% (798/3281) transcripts, were considered as unknown genes without any annotation in all the database used in shoots and roots, respectively (Table S4). Although these unknown genes might be novel genes in response to salt stress in peanut, but here we still paid more attention to investigate the expression patterns of the key gene families, which were reported involved in salt stress. To verify the RNA-seq data, 22 transcripts including 1-2 transcripts from each group listed Table 2 and partial from Table S4 were chosen to perform q-PCR analysis on mRNA level in shoots and/or roots. Almost of all DEGs investigated by q-PCR had similar expression patterns with the RNA-seq data (Figures S3, S4).

**Table 2:** Variation in transcripts in N4 and R3 treatments.

**Gene family**	**Transcript ID**	**Tissue**	**Relative abundance^a^**		
			**ST**	**N4**	**R3**
CNGC	CL252.Contig4	Root	2.12 ± 0.38	10.28 ± 1.75	7.49 ± 0.74
	Unigene22496	Root	10.10 ± 2.95	2.05 ± 1.08	1.23 ± 0.70
HKT1	CL7722.Contig1	Shoot	17.58 ± 3.16	3.80 ± 1.28	4.70 ± 0.58
	CL7722.Contig1	Root	15.84 ± 2.39	0.00 ± 0.00	0.26 ± 0.18
	CL7722.Contig2	Root	18.18 ± 2.80	1.20 ± 0.43	3.41 ± 0.78
H^+^-ATPases	CL10770.Contig2	Shoot	0.50 ± 0.11	44.21 ± 7.67	21.10 ± 4.62
	CL10770.Contig2	Root	2.38 ± 0.18	26.33 ± 0.99	4.83 ± 1.63
	CL10770.Contig3	Shoot	1.13 ± 0.39	52.81 ± 5.33	24.17 ± 4.92
	CL10770.Contig3	Root	4.17 ± 0.09	30.84 ± 4.17	4.88 ± 0.14
	Unigene18580	Shoot	1.16 ± 0.91	40.44 ± 16.69	13.18 ± 3.47
	Unigene18580	Root	2.18 ± 0.53	23.11 ± 3.07	3.68 ± 0.30
	Unigene18581	Shoot	1.08 ± 0.08	74.08 ± 10.57	35.45 ± 5.84
	Unigene18581	Root	2.82 ± 0.82	34.01 ± 2.74	6.09 ± 2.43
H^+^-pyrophosphatase	Unigene18887	Root	6.64 ± 3.38	11.18 ± 4.92	35.03 ± 4.15
K^+^ transporter	Unigene9700	Shoot	0.00 ± 0.00	12.59 ± 7.04	1.38 ± 0.60
	Unigene9701	Shoot	0.21 ± 0.25	14.45 ± 8.62	1.37 ± 2.25
LEA protein	CL7543.Contig2	Root	0.25 ± 0.09	0.27 ± 0.07	7.69 ± 2.35
	CL5798.Contig2	Root	243.04 ± 44.4	121.55 ± 13.1	68.99 ± 12.39
Photosynthesis related	CL9069.contig2	Shoot	0.04 ± 0.05	0.25 ± 0.25	0.25 ± 0.15
	Unigene23265	Shoot	69.67 ± 8.87	24.34 ± 10.18	27.28 ± 9.27
	Unigene21013	Shoot	119.87 ± 14.8	82.90 ± 4.83	83.39 ± 2.25
	Unigene25029	Shoot	225.51 ± 0.96	120.40 ± 10.7	120.66 ± 12.2
Aquaporin	Unigene15239	Shoot	0.09 ± 0.06	34.26 ± 23.08	8.14 ± 1.29
	Unigene15239	Root	0.19 ± 0.08	4.93 ± 0.60	1.40 ± 0.76
Proline metabolic related	CL3925.contig3	Shoot	21.03 ± 0.38	275.92 ± 35.1	145.14 ± 11.3
	CL3925.contig3	Root	3.84 ± 0.58	62.23 ± 6.27	22.51 ± 1.94
	CL3925.contig4	Shoot	1.49 ± 0.93	24.55 ± 7.27	7.72 ± 0.81
	CL11007.contig3	Shoot	0.14 ± 0.17	13.79 ± 8.51	3.85 ± 4.71
	CL11007.contig5	Shoot	7.44 ± 0.75	45.95 ± 12.53	27.70 ± 3.61
	Unigene16871	Shoot	27.31 ± 0.57	136.09 ± 26.5	92.17 ± 7.30
	Unigene16871	Root	27.94 ± 1.17	87.23 ± 18.37	72.84 ± 13.81
	Unigene33006	Root	0.82 ± 0.37	6.60 ± 0.79	15.56 ± 4.01

a * Relative abundance of transcripts was calculated from the RPKMs of three replicates in ST, N4, and R3 conditions, respectively*.

The entry of Na^+^ into plant cells is believed to be a passive process, and some candidate Na^+^ influx systems, such as non-selective cation channels (NSCCs), which were further divided into glutamate-activated channels (GLRs) and cyclic nucleotide gated channels (CNGCs) in *Arabidopsis*, have been proposed (Kaplan et al., 2007). It was reported that salt-tolerant peanuts showed good regulation of Na^+^ influx compared to the salt-susceptible varieties (Smitharani et al., 2014). In our data, we found 22 glutamate receptors, which were reported to possess extracellular-glutamate-gated ion channel activity, however, none of them responded to salt stress or the recovery treatment, in shoots or roots (Data not shown). We identified 48 CNGC transcripts, two of which were significantly different in salt-treated roots. The CL252.contig4 was 4.86-fold higher, while unigene22496 was 1/5 lower in the N4 treatment, but even lower in R3 (Table 2).

HKT is a group of Na^+^ transporters that has been shown to play a crucial role in the salinity stress tolerance of both monocotyledonous and dicotyledonous plants (Arshi et al., 2010). We identified two HKT genes AhHKT1;1 (CL7722.contig1) and AhHKT1;2 (CL7722.contig2) in DEGs, which were more homologous to HKT1 indicated by phylogenetic analysis. The expression of both genes was lower in plants under salt stress compared with ST, but slightly higher in R3 plants compared to N4 plants. AhHKT1;1 in N4 plant roots decreased to 1/1500 compared with that in ST (Table 2).

Sodium compartment by Na^+^/H^+^ antiporters powered by H^+^-ATPase or H^+^-pyrophosphatase provide an important mechanism to maintain a high K^+^/Na^+^ ratio when plants are under salt stress. We identified 20 Na^+^/H^+^ antiporter homologs from our transcriptome profiling, however, no variation were found in salt-stressed plants. Instead, we found nine plasma membrane H^+^-ATPases, which provide the driving force for the Na^+^ antiporters on plasma membrane, four of which (CL10770.contig2, CL10770.contig3, unigene18580, and unigene18581) were up-regulated in both shoots and roots under N4 conditions (Table 2 and Table S4). Only one H^+^-pyrophosphatase (unigene18887) in roots, which is considered to provide driving force for the vacuolar Na^+^ antiporters, did not changed by salt stress, but was 5.28-folds higher in R3 plants (Table 2). This implied that unigene18887 could play a role in Na^+^ efflux from vacuole when recovered from salt stress.

The intracellular potassium (K^+^) homeostasis is modulated by K^+^ channels and transporters. Two potassium (K^+^) transporters (unigene9700 and unigene9701) in shoots dramatically induced by more than 1200 times and 60 times in N4, respectively (Table 2). These data suggests that these genes play a role in keeping K^+^ homeostasis when plants grow in soils containing high Na^+^ concentrations.

Salt stress can cause the rapid accumulation of ROS, which perturb cellular redox homeostasis, damage membranes, and macromolecules. The major enzymatic antioxidants play roles in ROS detoxification system. Here we identified 98 varied enzymatic antioxidants including one ascorbate peroxidase (APX), one thioredoxin peroxidase, three peroxiredoxins (Prx), three alternative oxidases (AOX), four Superoxide dismutases (SOD), seven catalases (CAT), 33 peroxidases (POD), and 44 glutathione S-transferases (GST) from the DEGs. GSTs were identified as the largest group in response to salt stress and recovery in shoots and/or in roots. Expression of all GSTs was significantly greater under salt stress in either N4 or R3 compared to that in ST, and none were down-regulated (Table S2). In shoots, the expression of 25 GSTs was enhanced in N4, but only four of them in R3 plants were recovered to ST level. Eight GSTs in shoots had no response to salt stress, but were significantly elevated in R3 plants compared with ST plants. In roots, 19 GSTs were up-regulated in N4 plants, but all of them were recovered to ST level in R3. However, six root-specific expressed GSTs were identified, which were induced strongly by 10.33–504.95 times in N4 or R3 plants compared with ST plants (Table S4).

Late Embryogenesis Abundant (LEA) genes play key roles in alleviating the effects of active oxygen radicals and protecting cell membrane integrality. LEA proteins have been found in a wide range of plant species in response to water deficit resulting from desiccation, cold or osmotic stress. Among the DEGs, we found 14 LEA proteins, which belong to nine different groups according to Bies-Ethève et al. (2008). In shoots, 11 LEA genes were greatly up-regulated in N4 plants and only three of them in R3 plants recovered to ST level. However, three LEA transcripts were higher and one lower in N4 compared with ST in roots. In contrast to expectations, CL7543.contig2 and CL5798.contig2 in roots, which showed no response to salt stress, were induced by 30.93-folds and depressed to 28% in R3 compared with ST plants, respectively (Table 2 and Table S4).

We analyzed genes related to photosynthesis on the transcript level, including the genes coding the core protein subunits of photosystem I (PSI) and II (PSII) and ribulose-1, 5 bisphosphate carboxylase oxygenase (Rubisco). In the transcriptome data, 14, 11, 9, and 9 gene transcripts were identified as putative PSI subunits, PSII subunits, chlorophyll a/b-binding proteins, and Rubisco subunits, respectively (Table S5). Among these 43 genes, the expression of 29 transcripts was repressed to <60% in N4 compared with ST, yet most recovered to the ST level in R3. CL9069.contig2 in shoots was uniquely higher in both N4 and R3 conditions.

Plants produce more compatible solutes in response to osmotic stress. The functions of compatible solutes include maintaining cell turgor and thus the driving gradient for water uptake, stabilizing membranes, and proteins as free-radical scavengers or chemical chaperones. Proline is one of important amino acid compatible solutes. The transcriptome data showed that the delta-1-pyrroline-5-carboxylate synthetase (CL3925.contig3 in both shoots and roots and CL3925.contig4 and CL3925.contig5 in shoots) and pyrroline-5-carboxylate reductase (CL11007.contig3 and CL11007.contig5 in shoots), which catalyze the synthesis of proline, were significantly up-regulated in N4. Correspondingly, five proline dehydrogenase transcripts were down-regulated during salt stress in both shoots and roots, and all of them recovered to ST levels in R3. Paradoxically, two 1-pyrroline-5-carboxylate dehydrogenases (unigene16871 and unigene33006), which are the enzymes that catalyze the second step of proline degradation, were also up-regulated in both shoots and roots during salt stress (Table 2). In addition, although 5-oxoproline content, which could be involved in the glutathione and glutamate metabolism (Ohkama-Ohtsu et al., 2008), was also enhanced. But no 5-oxoprolinase genes, which play a role to hydrolyze 5-oxoproline to glutamate in an ATP-dependent reaction, were identified from the transcriptome data.

### Principal component analysis (PCA) and correlation analysis of plant physiological data, metabolites, and DEGs under salt stress and recovery conditions

**Figure 8:**
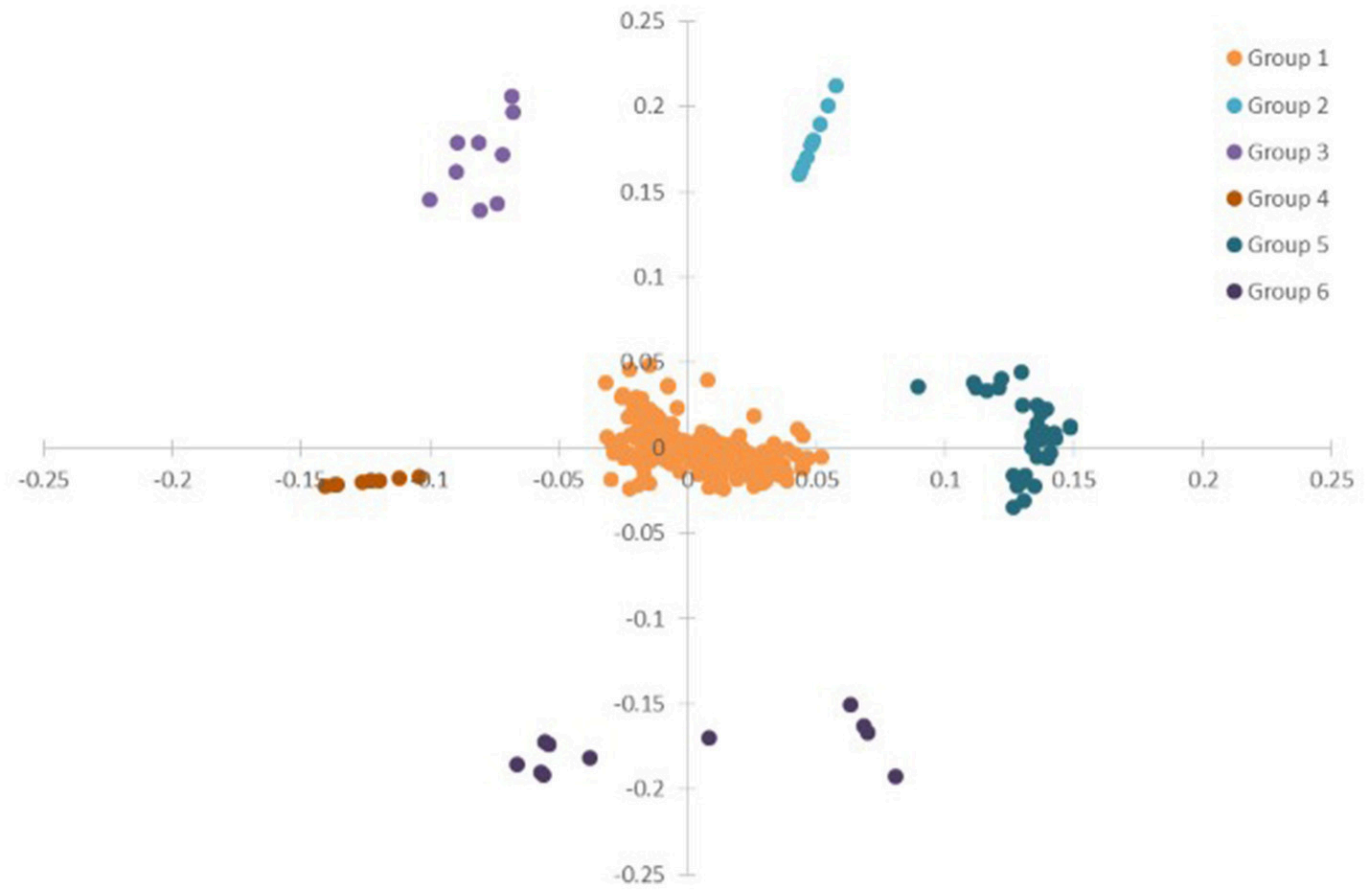
PCA of the physiological, transcriptome and metabolome data from ST, N4, and R3. ANOVA (with FDR correction) was applied on the physiological/transcriptome/metabolome data separately. And further the variables (physiological measurement/transcript/ metabolite) with FDR-corrected *p*-value < 0.001 were selected for the PCA based on the ANOVA analysis.

To have a global view of the links between the plant physiological parameters and variations of transcripts and metabolites, correlation analysis was done based on all the data from plant physiological analysis, metabolism, and DEGs in ST, N4, and R3 conditions. PCA analysis showed that the variables (physiological measurements/transcripts/metabolites) were divided into six clusters based on ANOVA with FDR correction (*p* < 0.001), which implies the variables in the same cluster might have co-variations in response to salt stress and recovery (Figure 8 and Table S6). Most (82.6%) variables were clustered in Group 1, including eight plant physiological parameters, 39 metabolites, and 333 transcripts. Ten metabolites were clustered in Group 2. The variation of 2-Amino-2-methylpropane-1,3-diol in shoot with 8 transcripts were clustered in Group 3. Nine and 11 transcripts were clustered in Group 4 and 6. Eight metabolites in shoots with 28 transcripts in roots were clustered in Group 5 (Figure 8 and Table S4).

### The inferred networks of metabolites and plant physiological and transcript variables

**Figure 9:**
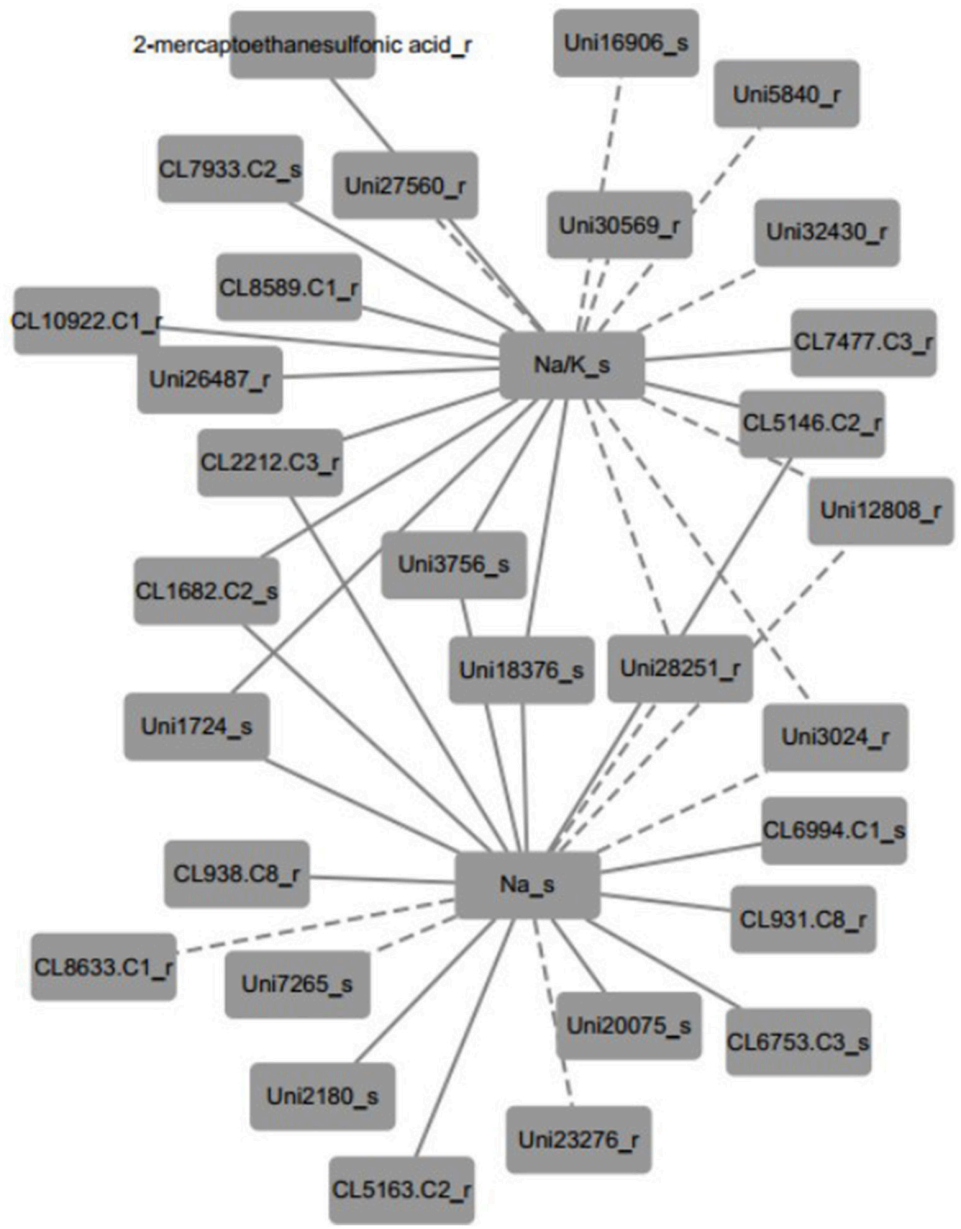
A representative inferred network of plant physiological, metabolite and transcript variables based on Pearson correlation (*p* < 0.001). Na^+^/K^+^ ratio in shoots was positively linked to 11 transcripts and 1 metabolite and negatively linked to 8 transcripts. Thirteen and six transcripts positively and negatively linked to Na^+^ content in shoots, respectively. Nine transcripts shared the same correlation with both Na^+^/K^+^ ratio and Na^+^ content in shoots. The solid edge represents positive correlation and the dashed edge represents negative correlation.

Furthermore, the inferred networks were declared by Pearson correlation analysis based on the data from plant physiology, metabolism, and transcriptome variation in N4 and R3 conditions (Figure 9 and Figure S5 and Table S7). Ninety-five transcripts and one metabolite, 28 of which were reported linked with salt stress and most were novel, were indicated as candidates involved in REC, Tr, Pn, and sodium and potassium in shoots or roots. A representative inferred network of Na^+^/K^+^_s and Na^+^_s related to metabolite and transcripts is shown (Figure 9). Eleven transcripts and 1 metabolite were indicated a positive correlation with Na^+^/K^+^ in shoots and 8 transcript variations have a negative correlation with Na^+^/K^+^ in shoots. Bile/Na^+^ symporter (CL7933.Contig2), Purple acid phosphatase 8-like (Unigene27560), Lysosomal Pro-X carboxypeptidase-like (CL1682.Contig2), Acidic endochitinase-like (Unigene3756), putative Cyclase family protein (Unigene18376), and 2 uncharacterized transcripts in shoots positively or negatively varied with Na^+^/K^+^ in shoots. Auxin induced PCNT115-like (CL8589.Contig1), LYSM RLK1-interacting kinase 1 like (CL10922.Contig1), EIN (ethylene insensitive) 4-like (Unigene26487), GPI ethanolamine phosphate transferase 2-like (Unigene30569), 2 CBL-interacting protein kinase like (Unigene5840 and Unigene28251), Insulin-degrading enzyme-like (CL2212.Contig3), cytochrome P450-like (CL5146.Contig2), and 3 uncharacterized transcripts in roots positively or negatively varied with Na^+^/K^+^ in shoots. Six transcripts positively and three negatively related to both Na^+^/K^+^_s and Na^+^_s in shoots were overlapped. In addition, seven transcripts including beta-Tubulin (CL938.Contig8_s), sarcosine oxidase (CL6753.Contig3_s), phosphate transporter (CL6994.Contig1_s), and 4 uncharacterized transcripts were positively linked to Na^+^_s and 3 transcripts including expansin A4-like (CL8633.Contig1_r), p-hydroxybenzoic acid efflux pump subunit (Unigene7265_s) and trihelix transcription factor GT-2-like (Unigene23276_r) were negatively linked to Na^+^_s. Furthermore, seven and four transcripts were positively and negatively to Pn, respectively. Only one transcript CL1183.contig2 in roots, which is a thioredoxin reductase, was positively linked with Tr. And REC was negatively linked with two transcripts (CL3136.contig2 and CL796.contig2 in roots), which are protein IQ-DOMAIN 14-like isoform 1 and myosin-H heavy chain-like, respectively. Seventeen transcripts including 6 positive and 11 negative, were correlated to K^+^ in shoots. Eighteen and 17 transcripts were linked to Na^+^/K^+^_r and Na^+^_r, respectively (Figure S4 and Table S7).

## Discussion

Soil drought or salinity is a transient condition depending on the water content in the soil, and studies showed that many aspects including water relation, ion balance, and gas exchange have involved during recovery course (Pardossi et al., 1998; Martre et al., 2002; Pérez-Pérez et al., 2007). Salt stress inhibits peanut growth (Singh et al., 2008; Salwa et al., 2010), but occasionally we observed that some plants would be wilt or even died after a heavy rain, which is in contrast to expectations. We mimicked these processes in the lab and found that leaf REC in the recovery conditions (R3) did not decrease, but increased further compared to the salt-stress treatment, implying that additional stress impaired the cell membrane stability (Figure 2B), however we do not found any related reports on this point. Further analysis suggested that Na^+^ in the roots could efflux back to the culture solution efficiently. However, the recirculation of Na^+^ from the shoots was rare in R3 conditions (Figure 2A), which is consistent with the results that leaf net Pn did not show any recovery in R3 (Figure 3A) and much more varied transcripts recovered in roots than that in shoots (Figure 5). It is consistent that Na^+^ and Cl^−^ in roots decreased faster than that in shoots in sorghum De Lacerda et al. (2005).

Plants accumulate a range of metabolites when exposed to salt stress. These metabolites serve as osmolytes and osmoprotectants, and protect plant cells under salt stress. The salt stress related metabolite profiling has been carried out in a subset of crops, such as soybean, barely, rice, chickpea, maize, and so on (Zhao et al., 2014; Dias et al., 2015; Nam et al., 2015; Richter et al., 2015; Shelden et al., 2016; Zhang et al., 2016). However, no such kind of research has been done in peanut till now. In this work, the KEGG pathway indicated several metabolism related pathways were strongly enriched (Figure 7 and Figure S2). And the metabolite profiling also revealed that the abundance of some metabolites were regulated by salt stress or salt stress recovery specifically (Table S4).

Plants have complex strategies to prevent the Na^+^ toxicity by limiting Na^+^ transport into shoots by SOS1 exporting Na^+^ from root tip and xylem and further sorting Na^+^ into the vacuole by NHX family members (Amtmann, 2009). Na^+^ concentration in shoots is approximant 40 mg g^−1^ DW in LH14, which is higher than that in *Thellungiella salsuginea* treated by 200–300 mM NaCl, but only half of that in *Arabidopsis thaliana* treated by 200 mM NaCl (Inan et al., 2004). However, in our study, most of the Na^+^ transporter related genes in peanut did not change significantly when plants were under salt stress, including the GLRs and CNGCs non-selective cation channels, which are proposed to be involved in the Na^+^ entry under salt stress (Kaplan et al., 2007; Kugler et al., 2009; Jin et al., 2014) and Na^+^/H^+^ antiporter, homologous to glycine max SOS1 and NHX families. But nine H^+^-ATPases on plasma membrane and one H^+^-pyrophosphatase on vacuole varied in both N4 and R3 conditions (Table 2). Thus, ATPases may play a major role in providing energy for Na^+^/H^+^ antiporters to maintain Na^+^ balance even though the abundance of antiporters did not change under salt stress conditions.

Plant behaviors in response to salt stress and recovery process could be attributed to the variation of their plant physiological parameter changes. Plant physiological parameter changes correlated with regulation of metabolites and proteins involved in salt stress and recovery process. PCA analysis was employed to identify metabolites and transcripts tightly correlated with plant physiology. Data showed that the variables (physiological parameters/transcripts/metabolites) were divided into six clusters based on ANOVA with FDR correction (*p* < 0.001), which imply the variables in the same cluster might have co-variations in response to salt stress and recovery (Figure 8 and Table S6). Pearson correlation analysis revealed several novel genes/transcripts positive or negative covariation with REC, transpiration, Na^+^ and K^+^ accumulation in shoots or roots (Figure 9). Further analysis the mechanisms of these genes/transcripts involved in salt stress and recovery process behind might improve our knowledge on the peanut salt stress and recovery response.

We profiled the data of peanut in response to salt stress and recovery from plant physiology, metabolism, and transcriptome. Among the 96 transcripts and metabolites related to REC, Tr, Pn, and sodium and potassium in shoots and roots were indicated, 28 of them have been reported involved in salt stress (Table S7). The metabolites and transcripts, inferred a role in salt stress or recovery in Figure 9 and Table 2, might worth more experiments to address their function in future studies. For instance, the salt stress strongly induced Unigene15239 (Table 2) is a like tonoplast intrinsic protein 3 (TIP3), which was aquaporin on vacuole membrane specifically expressed in seeds in other species and never reported induced by stresses in shoots or roots (Maurel et al., 1995). Considering cultivated peanut genome still unfinished, these work provides plenty of useful transcript information and candidate genes involved in salt stress and recovery for molecular and genetics researches in peanut.

## Author contributions

SW and GL planned and designed the research. FC, NS, YH and SL performed experiments. GD and YL collected data and conducted analysis. FC and GL wrote the manuscript.

### Conflict of interest statement

The authors declare that the research was conducted in the absence of any commercial or financial relationships that could be construed as a potential conflict of interest.

## Supplementary material

Figure S1 Cluster tree of all samples.

Figure S2 The mostly enriched KEGG pathway in shoots.

Figure S3 Real-time quantitative PCR analysis of partial DEGs in shoots.

Figure S4 Real-time quantitative PCR analysis of partial DEGs in roots.

Figure S5 The inferred networks of plant physiological, metabolite and transcript variables based on Pearson correlation (*p* < 0.001).

Table S1 The processed and normalized metabolite data.

Table S2 Metabolite variation data.

Table S3 R3 specific response metabolites.

Table S4 Transcript variation data.

Table S5 Photosynthesis relative gene variation.

Table S6 PCA data.

Table S7 Physiological parameter related transcripts and metabolites.

Table S8 Primers for q-PCR.

## Abbreviations

CDS: coding sequences
DEG: differentially expressed gene
GO: Gene Ontology
LEA: Late Embryogenesis Abundant
N4: 250 mM NaCl treated for 4 days
Nt: nucleotide
PCA: principal component analysis
Pn: net photosynthesis
PPFD: photosynthetic photon flux density
R3: recovery from N4 for 3 days
REC: relative electrolyte leakage
ST: standard conditions
Tr: transpiration
VIP: variable important in projection.
