# Supplementary figures and images for: Identification of Metabolites and Transcripts Involved in Salt Stress and Recovery in Peanut
Source: Front Plant Sci. 2018 Feb 22;9:217. doi: 10.3389/fpls.2018.00217 (PMC5827294; doi:10.3389/fpls.2018.00217)

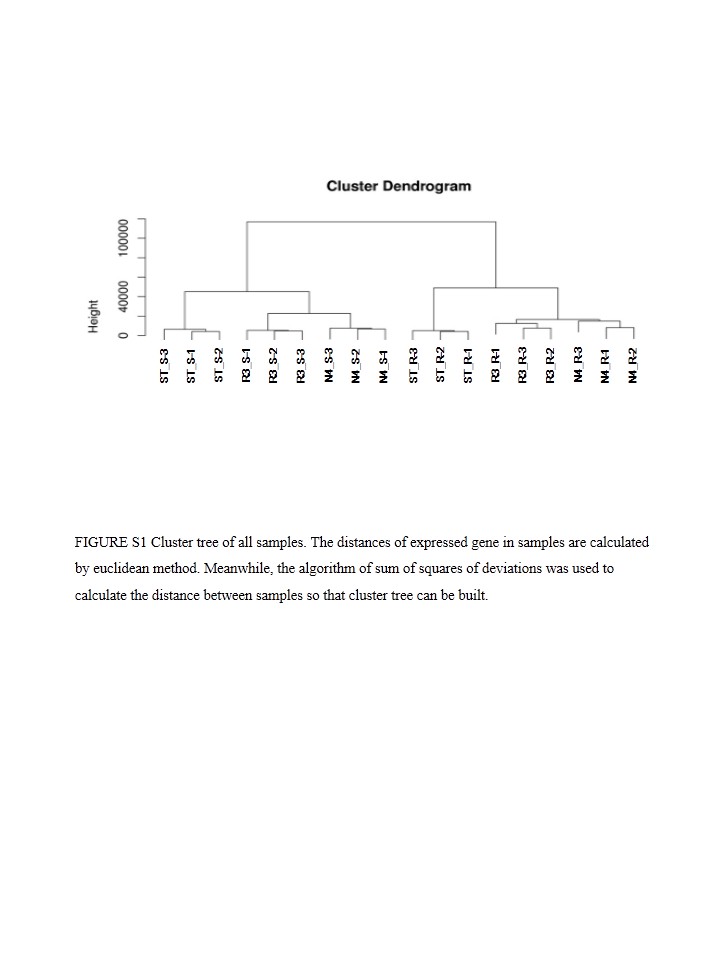

Supplement: Figure S1 — Cluster tree of all samples. [file Image1.JPEG]

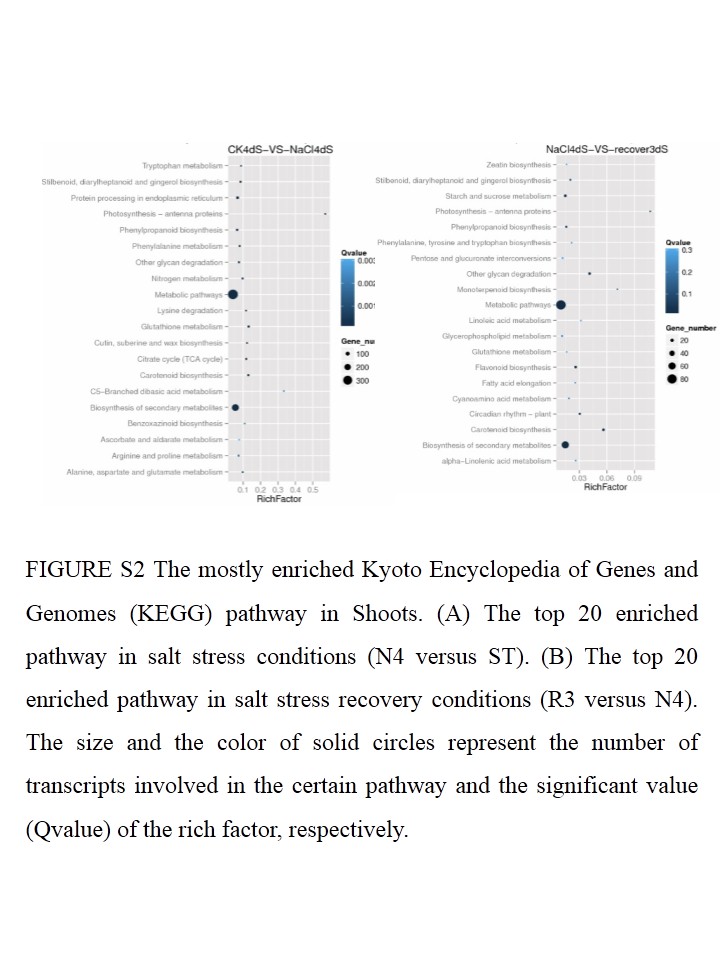

Supplement: Figure S2 — The mostly enriched KEGG pathway in shoots. [file Image2.JPEG]

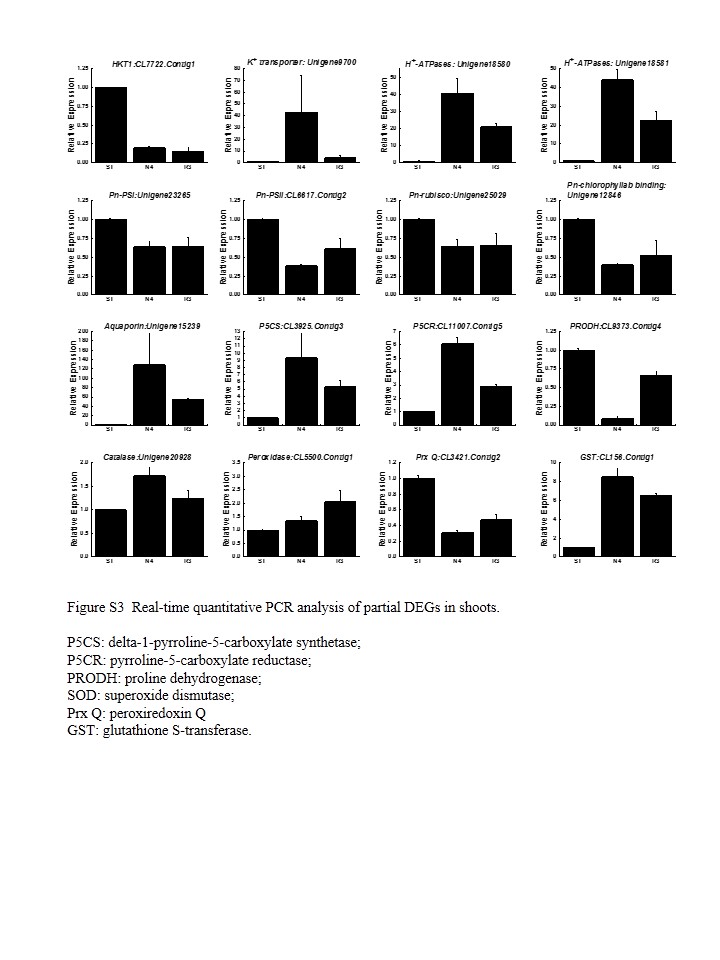

Supplement: Figure S3 — Real-time quantitative PCR analysis of partial DEGs in shoots. [file Image3.JPEG]

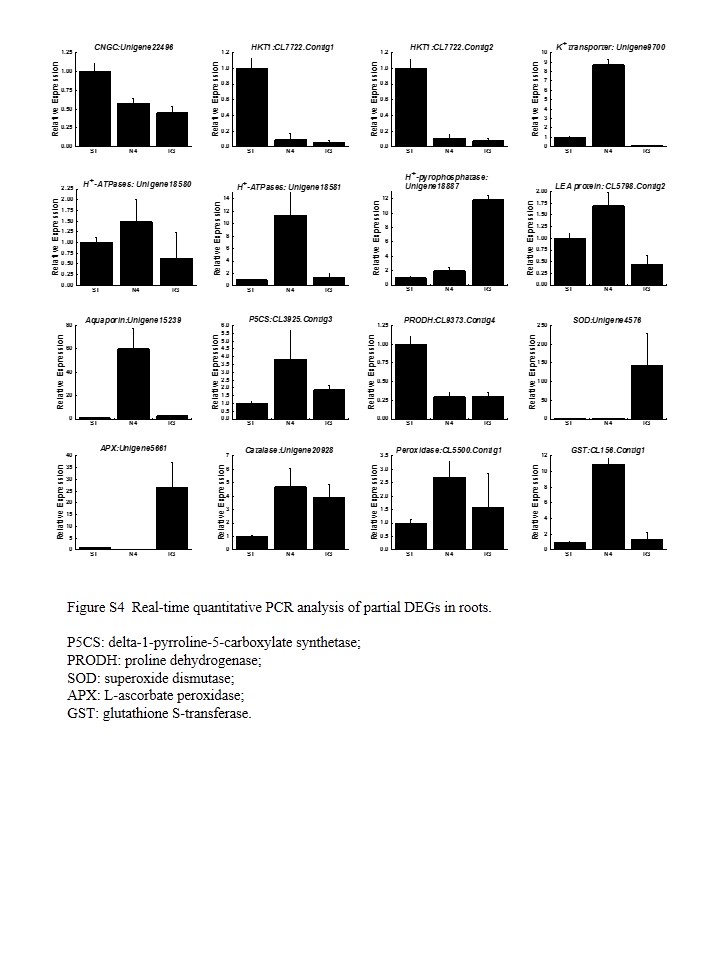

Supplement: Figure S4 — Real-time quantitative PCR analysis of partial DEGs in roots. [file Image4.JPEG]

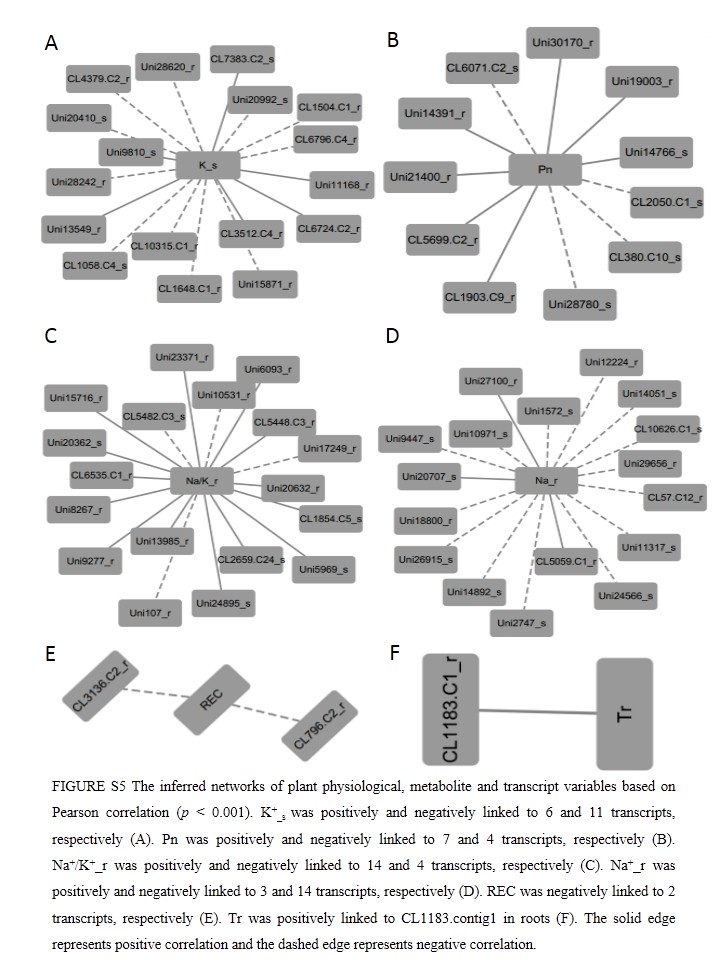

Supplement: Figure S5 — The inferred networks of plant physiological, metabolite and transcript variables based on Pearson correlation (p < 0.001). [file Image5.JPEG]
